# Supplementary material for: Towards Novel Fluorinated Methacrylic Coatings for Cultural Heritage: A Combined Polymers and Surfaces Chemistry Study
Source: Polymers (Basel). 2019 Jul 16;11(7):1190. doi: 10.3390/polym11071190 (PMC6681005; doi:10.3390/polym11071190)
Supplement: Supplementary file 1 [file polymers-11-01190-s001.pdf]

Supporting Information

Towards novel fluorinated methacrylic coatings for Cultural Heritage: a combined Polymers and Surfaces Chemistry study

Valentina Sabatini[1,2,*], Eleonora Pargoletti[1,2], Valeria Comite[1], Marco Aldo Ortenzi[1,2,3], Paola Fermo[1], Davide Gulotta[4,5] and Giuseppe Cappelletti[1,2,3]

[1] Dipartimento di Chimica, Università degli Studi di Milano, Via Golgi 19, 20133, Milano, Italy; valentina.sabatini@unimi.it; eleonora.pargoletti@unimi.it; valeria.comite@unimi.it; marco.ortenzi@unimi.it; paola.fermo@unimi.it; giuseppe.cappelletti@unimi.it

[2] Consorzio Interuniversitario per la Scienza e Tecnologia dei Materiali (INSTM), Via Giusti 9, 50121, Firenze, Italy;

[3] CRC Materiali Polimerici "LaMPo", Dipartimento di Chimica, Università degli Studi di Milano, Via Golgi 19, 20133, Milano, Italy;

[4] The Getty Conservation Institute, 1200 Getty Center Drive, Suite 700, Los Angeles, CA; dgulotta@getty.edu

[5] Dipartimento di Chimica, Materiali e Ingegneria Chimica "Giulio Natta", Politecnico di Milano, Via Mancinelli 7, 20131, Milano.

* Correspondence: valentina.sabatini@unimi.it ; Tel.: +39-02-503-14115

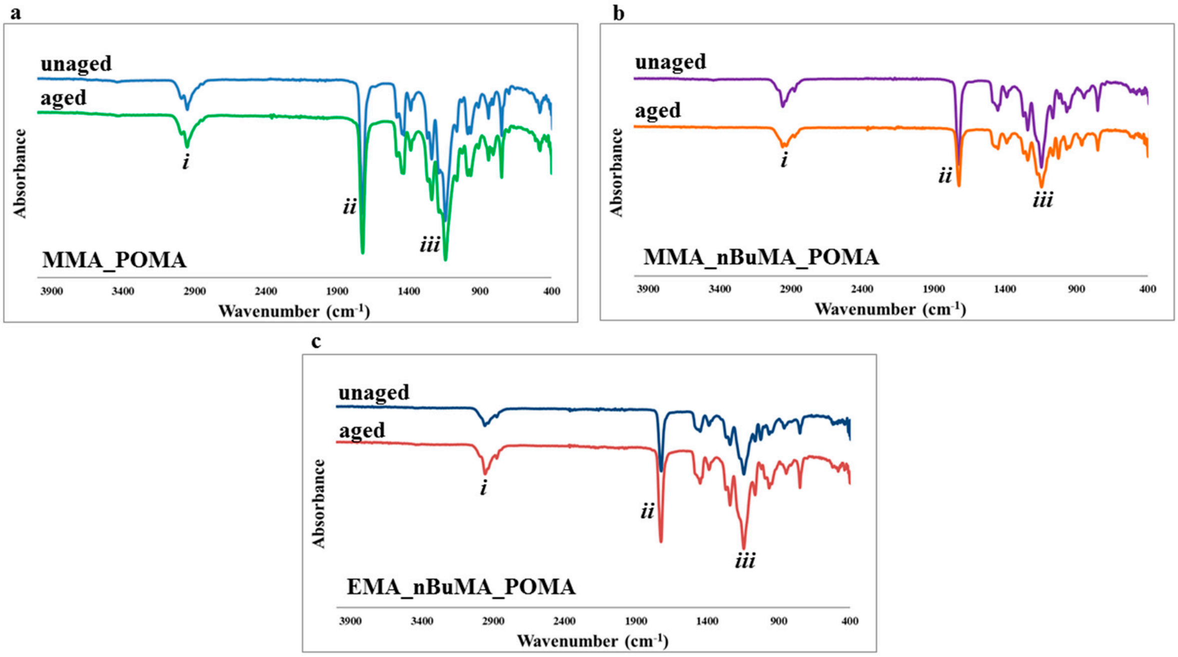

Figure S1. FT-IR spectra before and after the UV aging test of a) MMA_POMA, b) MMA_nBuMA_POMA and c) EMA_nBuMA_POMA samples.

Figure S1 reports a correlation between FT-IR spectra of POMA-based samples before and after the UV aging test. The peak (i) in the ~3100-2800 cm^{-1} range is related to the bending of C-H aliphatic bonds; the stretching of carbonyl ester groups (C=O) (ii) appears between ~1750-1600 cm^{-1} and the characteristic absorption band for the symmetric stretching vibration of C-O conjugated to carbonyl ester groups (iii) lies in the range ~1350-1100 cm^{-1}. In the case of MMA_POMA resin subjected to UV aging, the shape of the three absorption peaks considered as reference remains unchanged; on the other hand, for nBUMA-based aged resins, it is possible to observe a small change in terms of peaks intensity in the area between 1350 and 1100 cm^{-1}, related to the breaking of carbonyl esters bonds.

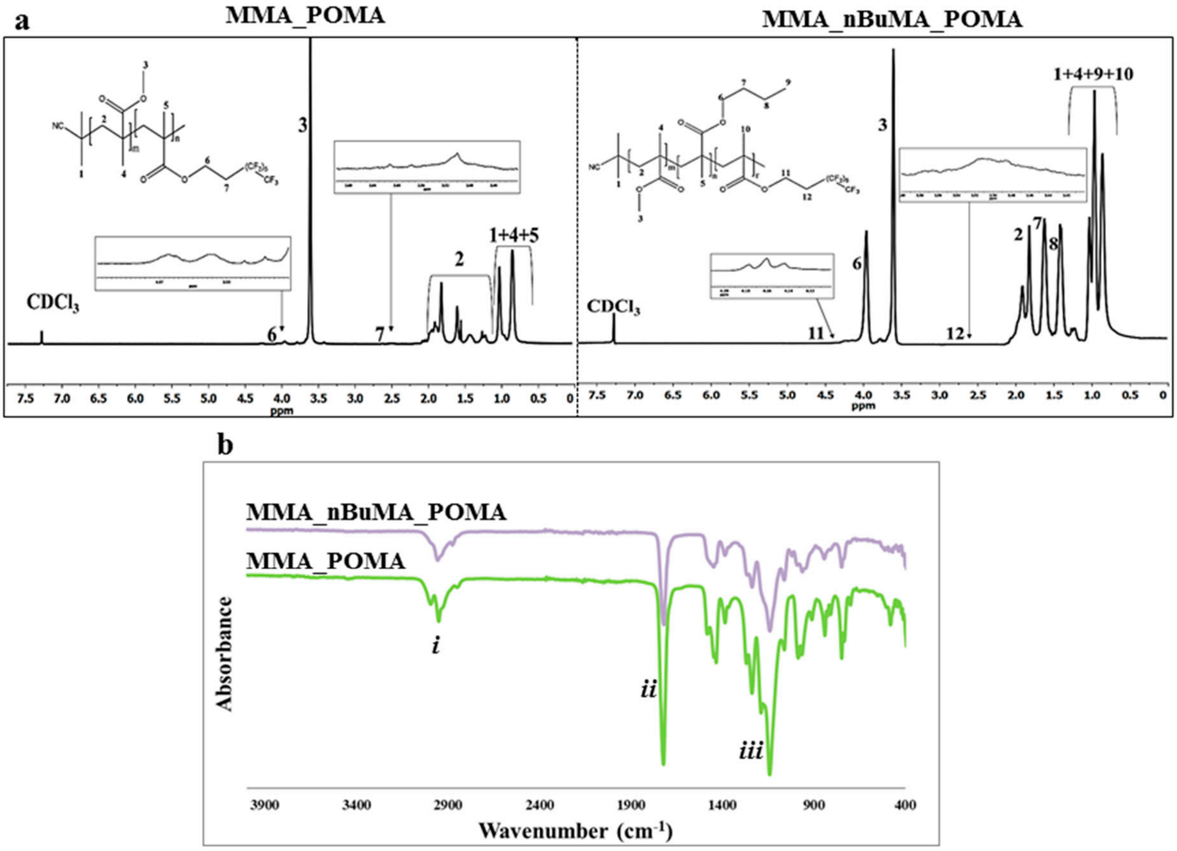

Figure S2. ^{1}H NMR and FT-IR spectra of a) MMA_POMA and b) MMA_nBuMA_POMA resins removed from stone substrates.

Figure S2 shows MMA_POMA and MMA_nBUMA_POMA a) ^{1}H NMR spectra and b) FT-IR curves collected after removal. From the comparing of unaged (Figure 3 and S1) and aged macromolecular and chemical structures, it is worth of note the absence of significant differences in terms of chemical bonds and peaks intensity change of the resulting polymers.
